# Central nervous system insulin signaling can influence the rate of insulin influx into brain

**DOI:** 10.1186/s12987-023-00431-6

**Published:** 2023-04-19

**Authors:** Van Nguyen, Peter Thomas, Sarah Pemberton, Alice Babin, Cassidy Noonan, Riley Weaver, William A. Banks, Elizabeth M. Rhea

**Affiliations:** 1 School of Medicine, University of Washington, Seattle, WA 98195 USA; 2 Geriatric Research Education and Clinical Center, Veterans Affairs Puget Sound Health Care System, Seattle, WA 98108 USA; 3 University of Washington, Seattle, WA 98195 USA; 4 Department of Medicine, Division of Gerontology and Geriatric Medicine, University of Washington, Seattle, WA 98195 USA

**Keywords:** Blood-brain barrier, Insulin receptor, Insulin resistance, Alzheimer’s disease

## Abstract

**Background:**

Insulin transport across the blood-brain barrier (BBB) is a highly regulated, saturable process, known to be affected by many peripheral substrates including insulin itself and triglycerides. This is in contrast to insulin leakage into peripheral tissues. Whether the central nervous system (CNS) can control the rate of insulin uptake by brain remains to be determined. Insulin BBB interactions are impaired in Alzheimer’s disease (AD) and CNS insulin resistance is widely prevalent in AD. Therefore, if CNS insulin controls the rate of insulin transport across the BBB, then the defective transport of insulin seen in AD could be one manifestation of the resistance to CNS insulin observed in AD.

**Methods:**

We investigated whether enhancing CNS insulin levels or induction of CNS insulin resistance using an inhibitor of the insulin receptor altered the blood-to-brain transport of radioactively labeled insulin in young, healthy mice.

**Results:**

We found that insulin injected directly into the brain decreased insulin transport across the BBB for whole brain and the olfactory bulb in male mice, whereas insulin receptor blockade decreased transport in female mice for whole brain and hypothalamus. Intranasal insulin, currently being investigated as a treatment in AD patients, decreased transport across the BBB of the hypothalamus.

**Conclusions:**

These results suggest CNS insulin can control the rate of insulin brain uptake, connecting CNS insulin resistance to the rate of insulin transport across the BBB.

## Introduction

Insulin blood-brain barrier (BBB) transport is critical for delivering insulin to the brain. Insulin has multiple roles in the brain, regulating metabolism and cognition [1]. In conditions, including obesity and Alzheimer’s disease (AD), where there is a dysfunction in brain insulin action, known as insulin resistance, insulin BBB transport is also impaired [2]. Therefore, it is clear there is a relationship between insulin BBB transport and brain insulin resistance. Understanding more about the regulation of insulin transport into the brain will help us understand how brain insulin resistance arises and suggest therapeutic targets for prevention.

Insulin levels in the cerebrospinal fluid (CSF) are approximately 1/10th that in the blood under baseline conditions [3–5]. Insulin can be detected in the interstitial fluid and is similar to levels in the CSF [3]. Historical radioactive binding assays suggest insulin is present throughout the entirety of the brain [6] and immunoactive measurements of insulin have been assessed in the rat brain [7]. The olfactory bulb and hypothalamus contain some of the highest levels of insulin, while the amygdala and midbrain have much less [7]. Insulin is predominantly made in the pancreatic β cells and released into the circulation. Recent evidence suggests insulin may be synthesized and released in small amounts within the choroid plexus [8]. However, BBB transport is still considered the primary regulator of insulin availability within the brain and therefore, must have appropriate mechanisms in place to transport insulin into the brain. This contrasts with tissues in the periphery, which lack the unique structure of the BBB, and have greater access to the availability of insulin due to more porous endothelial barriers, including the liver and kidney. Muscle delivery of insulin does require navigating a tighter endothelial barrier than other peripheral tissues and this process is still under active investigation [9].

Insulin BBB transport has been investigated for multiple decades [6, 10]. Insulin transport is known to be saturable [11, 12], unidirectional from blood-to-brain [13], selective for insulin [14] although the degree of specificity is currently unknown, conserved across species [11, 12, 15, 16], has a varying rate of transport among brain regions [6, 17], and requires intact insulin [18, 19]. Additionally, insulin BBB transport occurs independent of the endothelial insulin receptor as recently shown by our group [20] and confirmed by others [21–23]. Lastly, insulin BBB transport is modified by many serum factors [11, 19] and in various diseases and conditions, including obesity, diabetes, AD, aging, and exercise [2, 6, 10, 24]. However, whether the brain can control its own rate of insulin BBB transport has not been directly tested.

Intranasal (IN) insulin therapy is currently being investigated in the treatment of cognitive impairment in AD [25]. This route of delivery is known to bypass the BBB and delivers insulin directly to the brain via the cribriform plate, without affecting peripheral glucose levels [26]. In the current study we wanted to identify whether the therapeutic delivery of IN insulin could affect the endogenous insulin transport system across the BBB. The distribution of insulin following IN delivery differs compared to the endogenous route for insulin to access the brain and from distribution after intracerebroventricular (ICV) injection [26]. Therefore, it is important to understand whether IN insulin therapy could alter the BBB transport of insulin.

Using young, healthy male and female CD-1 mice, we investigated radioactive insulin blood-to-brain transport following brain manipulation of insulin signaling. We utilized various methods involving IN and ICV administration to compare and contrast the two routes of delivery [26]. We also investigated whether activation of brain insulin signaling or inhibition of insulin receptor signaling, as occurs in brain insulin resistance, could impact insulin BBB transport by delivering insulin or the selective insulin receptor antagonist S961 [27], directly to the brain.

## Methods

### Animals

Two-month-old male and female CD-1 mice (Charles River Laboratory, Seattle, WA) were kept on twelve-hour light/dark cycles (lights on at 6:00 AM) and provided *ad libitum* access to food and water. CD-1 mice are an established model for BBB transport studies. To minimize pain and distress, all mice were anesthetized with an intraperitoneal injection of 0.15 mL of 40% urethane before each study and kept on a heating pad to maintain normal body temperature. All animal procedures were approved by the VA Puget Sound Health Care System Institutional Animal Care and Use Committee and implemented under guidelines and regulations at a facility certified by the Association for Assessment and Accreditation of Laboratory Animal Care International (AAALAC).

### Radioactive labeling

The use of radioactive tracers allows quantifying BBB pharmacokinetics without the need to administer high doses of insulin necessary for immunoactive measurements. One millicurie (mCi) of ^125^I (Perkin Elmer, Waltham, MA, USA) was added to 10 µg of human insulin (Sigma-Aldrich St Louis, MO, USA) or S961 (gift from Novo Nordisk, Denmark) diluted in 0.25 M chloride-free sodium phosphate buffer (PB, pH 7.5). The first step involved adding 10 µg chloramine-T (Sigma-Aldrich) diluted in 0.25 M PB to initiate the radioactive labeling reaction for a final reaction volume of 60 µl. After 60 s, 100 µg of sodium metabisulfite (Sigma-Aldrich) in 10 µl was added to terminate the reaction. Albumin (Sigma-Aldrich) was labeled with Technetium-99 m (^99m^Tc, Radioisotope Life Sciences, Tampa, FL, USA) by combining 1 mCi of ^99m^Tc, 1 mg bovine serum albumin (BSA) with 0.5 ml deionized water, 120 µg stannous tartrate, and 20 µl 1 M HCl for 20 min. To purify both ^125^I-insulin and ^99m^Tc-albumin, a column of Sephadex G-10 (Sigma-Aldrich) was used, and 100 µl fractions were collected in 100 µl 1% BSA/lactated Ringers (BSA/LR). Protein binding to the respective isotope was characterized by 30% trichloroacetic acid (TCA) precipitation. The radioactively labeled substrates used were quality-controlled, negating the overestimation of free radioactivity. The estimated specific activity for ^125^I-insulin has been previously calculated to be 55 Ci/g [11].

### ICV delivery and distribution

To measure the distribution of ^125^I-insulin and ^125^I-S961 following ICV delivery, we used methods previously described for other regulatory peptides [28, 29]. The scalp was reflected in anesthetized mice and a hole was drilled 1 mm lateral and 0.2 mm posterior to the Bregma. A 26 G Hamilton syringe was used to inject 1 µl of ^125^I-insulin or ^125^I-S961 (25,000 CPM in 1% BSA/LR) at a depth of 2.5 mm into the right ventricle of the brain. Brains were removed between 2 and 60 min later and dissected into regions. Blood was collected from the left carotid artery and spun at 3,200xg for 10 min. Weighed brain regions and serum were counted in a gamma counter (Wizard2, Perkin Elmer, Waltham, MA, USA). The amount of radioactivity detected in each region (CPM/region) or serum (CPM/ul serum) was divided by the amount injected (CPM/Inj) and multiplied by 100 to calculate the %Inj. The %Inj was divided by the weight of the region (g/region) or the volume serum measured (ul/serum) to calculate the %Inj/g and %Inj/ml. Pilot studies were performed to identify ventricular injection using this method.

### BBB pharmacokinetics - multiple-time regression analysis (MTRA)

For all radioactive studies, the right jugular vein and left carotid artery were exposed. A radioactive intravenous (IV) injection of 0.2 ml of 1% BSA/LR containing 1 × 10^6^ CPM of ^125^I-insulin and 5 × 10^5^ CPM of ^99m^Tc-albumin was injected into the right jugular vein, with ^99^Tc-albumin acting as a marker for vascular space. For studies involving ICV injections (method described above), mice first received a 1 µl ICV injection of vehicle (PB), human insulin (1 µg in PB), or the insulin receptor antagonist, S961 (1 µg in PB). Mice were injected IV with the radioactive tracer 10 min later. For studies involving IN injections, mice received a 1 µl injection of vehicle (1% BSA/LR) or insulin (1 µg in 1% BSA/LR) at the level of the cribriform plate as previously described [30]. At a time when insulin levels are highest in the brain following IN insulin injection (30 min later) [30], mice received an IV injection containing the radioactive tracer. Blood was collected from the left carotid artery between 0.5 and 10 min after IV injection, followed by immediate decapitation. The whole brain, olfactory bulb, and in some cases the hypothalamus were removed and weighed. Serum was collected by centrifuging the arterial blood at 3,200xg for 10 min. Each mouse yielded a single serum sample and time-matched brain sample. Exposure time was calculated from the formula:

1 $$Exposure \; time= \frac{{\int }_{0}^{t}Cp\left(t\right)dt}{Cp\left(t\right)}$$,

where *Cp* is the level of radioactivity (CPM) in serum at time (*t*). This value corrects for the clearance of insulin from the blood. Insulin influx was calculated by MTRA:

2 $$\frac{Am}{Cpt}=Ki \left(\frac{{\int }_{0}^{t}Cp\left(t\right)dt}{Cp\left(t\right)}\right)+Vi$$,

where *Am* is the level of radioactivity (CPM) per gram of brain tissue at time (*t*), *Cpt* is the level of radioactivity (CPM) per ml arterial serum at time *t*, *K*_*i*_ (µl/g-min) is the steady-state rate of unidirectional solute influx from blood to brain. *V*_*i*_ (µl/g) is the level of solute in rapid, reversible equilibrium between plasma and brain. Subtracting ratios for ^99m^Tc-albumin from the corresponding ^125^I-insulin brain/serum (B/S) ratio yielded a delta B/S ratio, which corrects the B/S ratio for ^125^I-insulin for vascular space. The linear portion of the relation between the delta B/S ratio versus exposure time is represented in the figures and was used to calculate the *K*_*i*_ (µl/g-min) with its standard error term, and the y-intercept determined representing the *V*_*i*_(µl/g).

### Regional distribution

For the regional studies, brain and serum were collected at a single time point post central nervous system (CNS) manipulation. For IN studies following ICV delivery, S961 (1 µg in PB) or vehicle was given ICV, and IN ^125^I-insulin was immediately administered. 30 min later, brain and serum were collected. For IV studies following IN delivery, insulin (1 µg in PB) or vehicle was given IN, and IV ^125^I-insulin was administered 30 min later. For IV studies following ICV delivery, S961 (1 µg in PB) or vehicle was given ICV and 10 min later, IV ^125^I-insulin and ^99m^Tc-albumin was injected. For the IV studies, brain and serum were collected 5 min later. Olfactory bulb (OB) and the whole brain dissected into 10 regions [31] were weighed: striatum (St), frontal cortex (FC), hypothalamus (Hy), hippocampus (HC), thalamus (Th), parietal cortex (PC), occipital cortex (OC), cerebellum (CB), midbrain (MB), and pons-medulla (PM). Whole brain (WB) is the summed value from individual regions. The levels of radioactivity in serum (50 µl) and brain samples were counted in a gamma counter (Wizard2, Perkin Elmer, Waltham, MA, USA). %Inj/g for IN distribution and Delta B/S ratios following IV administration were calculated as described above. ^99m^Tc-albumin B/S ratios are also presented to identify changes in regional vascular space following CNS manipulation.

### Immunoactive insulin measurement

Blood was collected from the descending aorta in anesthetized mice 30 min following ICV insulin (1 µg) or ICV S961 (1 µg). Whole blood was centrifuged at 3,200xg for 10 min. Serum was collected, aliquoted, and frozen at -80 °C until measurement. Mouse insulin was measured in 1:3 diluted serum using the single-plex Meso Scale Discovery mouse insulin kit (MSD, Rockville, MD) according to the manufacturer’s directions. This kit cross reacts with human insulin.

### Statistical analysis

Regression analyses and other statistical analyses were performed with Prism 9.0 (GraphPad Softward Inc., San Diego, CA, USA). For all MTRA pharmacokinetic studies, the slope of the linear regression lines (K_i_), reported with their correlation coefficients (r), and y-intercepts (V_i_) were compared statistically using Prism equivalent to an analysis of covariance (ANCOVA) [32]. For these studies, the individual data points are presented in the representative Figures. The linear regression data, correlation coefficients, and statistical results are presented in the Tables. For the regional studies, the regional B/S levels for each sex were reported with their standard error terms and analyzed by a two-way analysis of variance (ANOVA) followed by Tukey’s post hoc test when appropriate. A student’s t test was used when immunoactive levels of insulin were compared between two groups.

## Results

### Distribution of ^125^I-insulin and ^125^I-S961

**Fig. 1:**
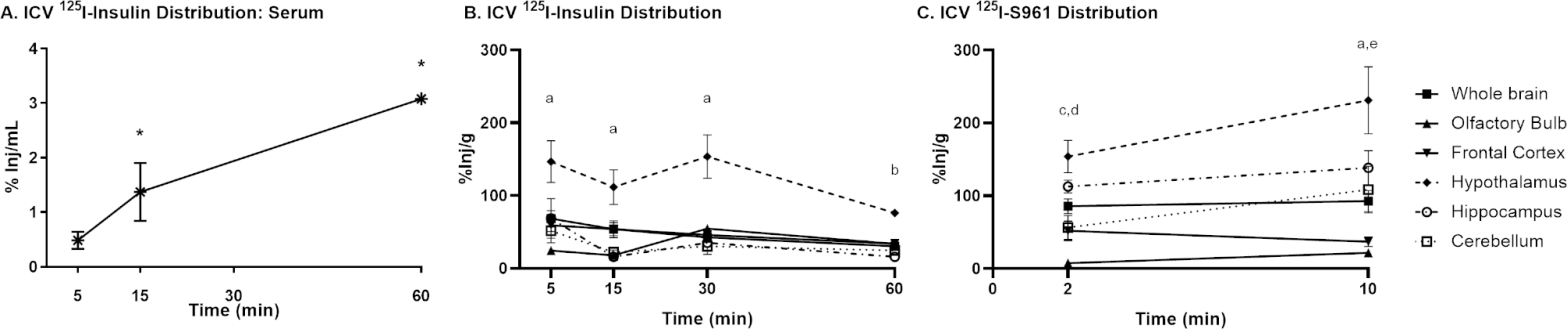
Distribution of ^125^I-insulin and ^125^I-S961 following ICV delivery. Distribution of **(A)**^125^I-insulin in serum and **(B)**^125^I-insulin or **(C)**^125^I-S961 throughout the brain was measured over time. There were significant differences over time for serum (one-way ANOVA *p < 0.05) and between brain regions (two-way ANOVA: time and region p < 0.05; post hoc analysis as marked: ^a^p<0.05 hypothalamus vs. all other regions; ^b^p<0.05 hypothalamus vs. hippocampus; ^c^p<0.05 hypothalamus vs. olfactory bulb, frontal cortex, and cerebellum; ^d^p<0.05 olfactory bulb vs. hippocampus, ^e^p<0.05 hippocampus vs. olfactory bulb and frontal cortex; n = 2–4 /time point)

First, we identified distribution and possible site of action for human insulin and S961. We have shown following IN administration that ^125^I-insulin distributes throughout the brain over time to all regions and peaks after about 30 min in male CD-1 mice [30]. We show here that following ICV delivery into the lateral ventricle of the brain, ^125^I-insulin and ^125^I-S961 also distribute throughout the brain. Within 5 min ^125^I-insulin appears in the serum and significantly increases with time, to about 2%Inj/ml by the 30 min time point (Fig. 1A). As this is about 1/10th of the level of insulin seen after IV injection, we can conclude that most of the insulin administered ICV does not enter the blood stream. ^125^I-insulin rapidly spreads to regions of the brain including the hypothalamus, cerebellum, and olfactory bulb (Fig. 1B). Levels in the hypothalamus remain high up until 60 min and levels in other regions plateau after 15 min. Within 2 min after ICV injection, ^125^I-S961 has rapidly spread to regions of the brain, including the hypothalamus (Fig. 1C). Levels in the hypothalamus continue to increase up until 10 min and levels in other regions remain at a plateau after 2 min. These data and our previous data [30] suggest that ^125^I-insulin is distributed throughout the brain following IN or ICV delivery within 5 min. ICV ^125^I-S961 is also distributed throughout the brain within 2 min.

### Effect of CNS treatment on ^125^I-insulin serum clearance

**Fig. 2:**
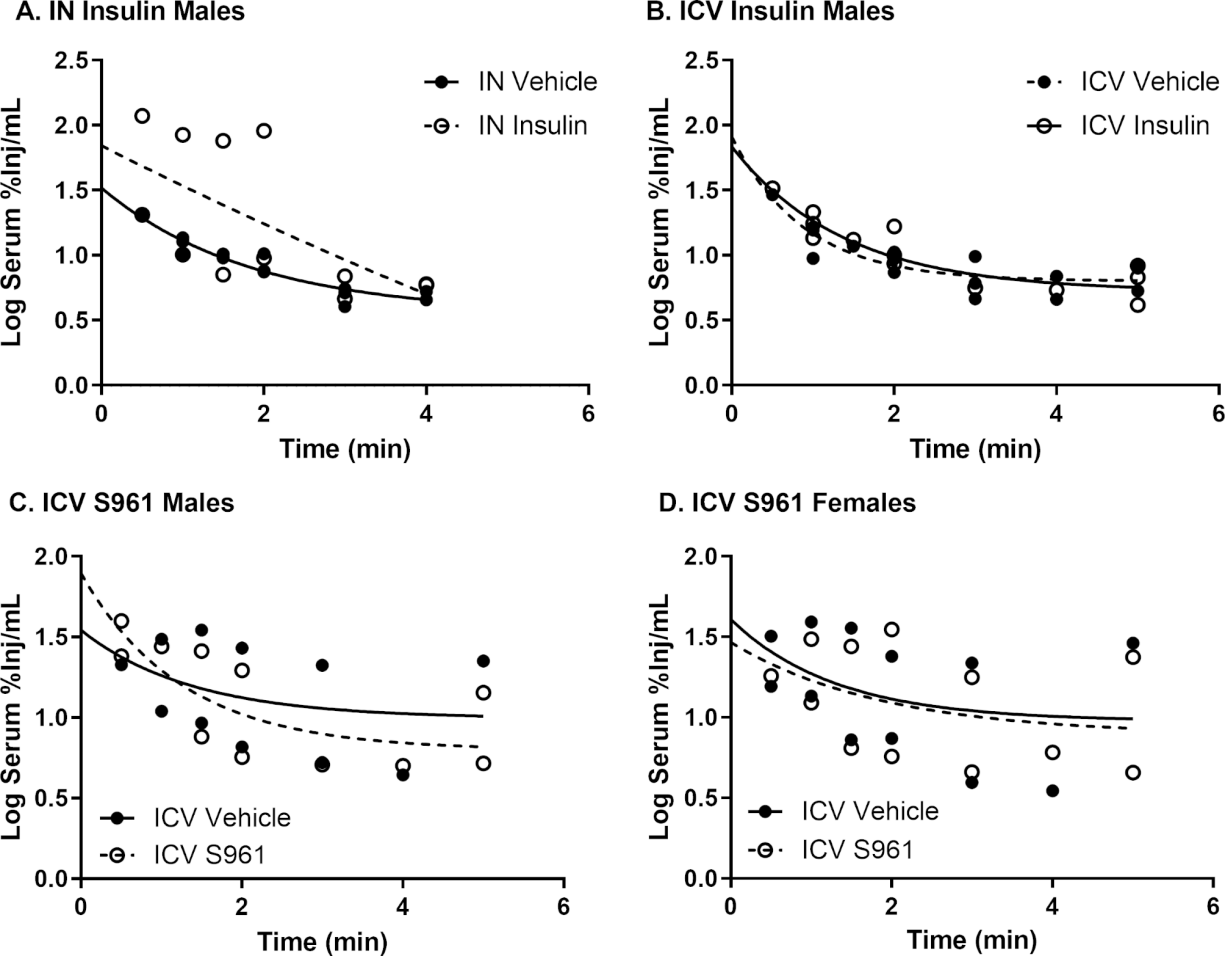
^125^I-insulin serum kinetics following CNS treatment. There was no difference in ^125^I-insulin kinetics following **(A)** IN insulin in males, **(B)** ICV insulin in males, **(C)** ICV S961 in males, or **(D)** ICV S961 in females. Each data point represents a single mouse

In order to investigate insulin brain uptake, we first needed to identify whether CNS treatment with insulin or S961 could affect ^125^I-insulin serum clearance. To do these studies, we assessed the effects of administering 1 µg IN or ICV of human insulin or 1 µg ICV of S961 on the clearance of intravenously administered ^125^I-insulin. The blood level of ^125^I-insulin was assessed within 5 min after its IV injection and 30 min after the IN administration or 10 min after the ICV administration of insulin or S961, a time when we have shown these substances are distributed throughout the CNS. We identified there was no effect of IN or ICV insulin on serum clearance in male mice (Fig. 2A, B). Additionally, there was no effect of ICV S961 on ^125^I-insulin serum clearance in male or female mice (Fig. 2C, D). These data suggest there was no direct effect on ^125^I-insulin serum clearance following CNS treatment with the doses of insulin or S961 used in the time frame investigated.

### Effect of CNS treatment on BBB permeability and brain vascular space

**Fig. 3:**
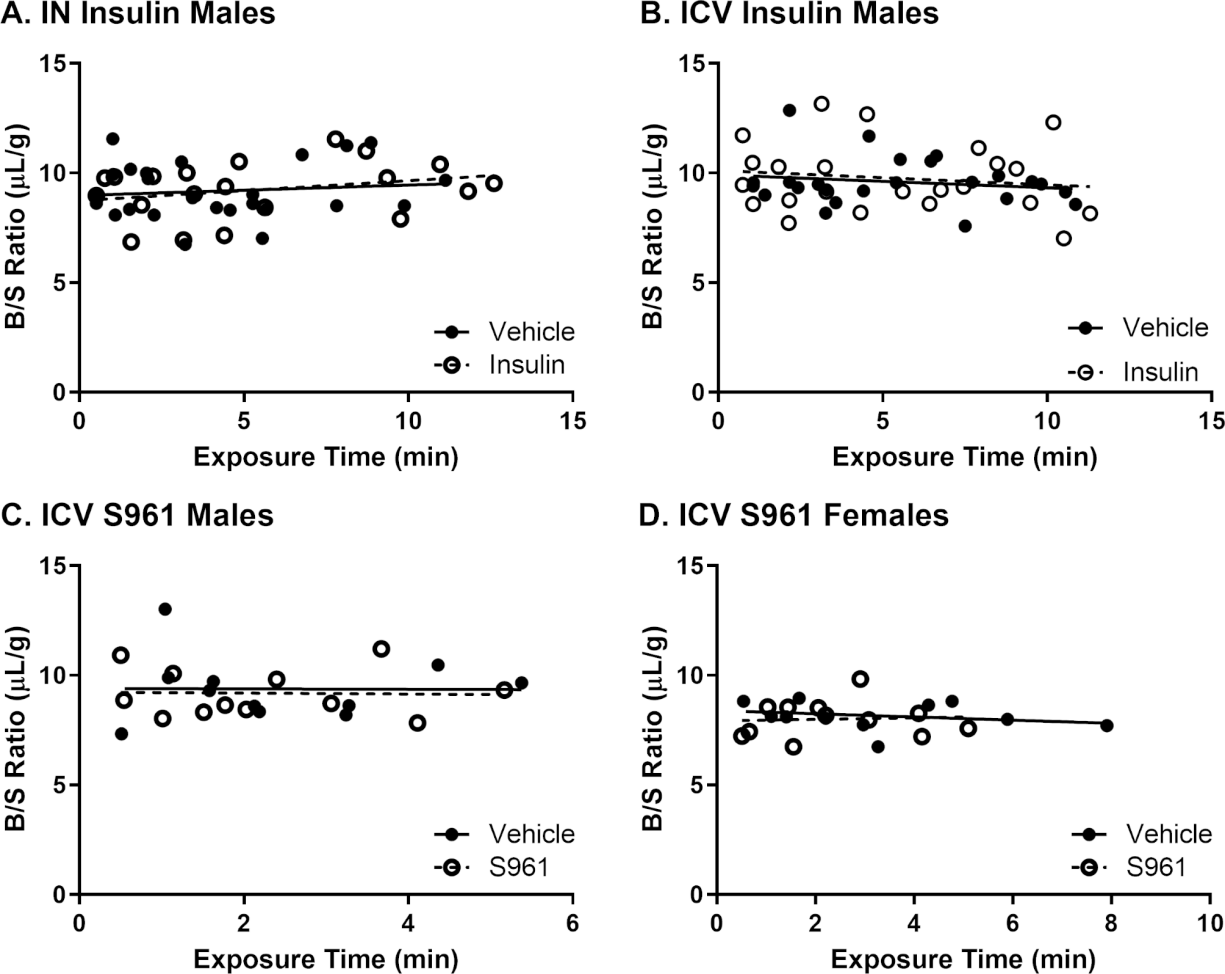
Permeability of the whole brain BBB to ^99m^Tc-albumin following CNS treatment. There was no significant multiple-time linear regression in ^99m^Tc-albumin Brain/Serum (B/S) ratios following **(A)** IN insulin in males, **(B)** ICV insulin in males, **(C)** ICV S961 in males, or **(D)** ICV S961 in females, indicative of no transport of this vascular marker within the time frame investigated. Each data point represents a single mouse

We then determined that ICV or IN insulin or ICV S961 had no effect on whole brain vascular space or BBB leakage as measured with ^99m^Tc-albumin (Fig. 3). The whole brain B/S ratios are typical for what we normally observe [20] in these types of transport studies (range of 8–12 µl/g). Additionally, there was no significant linear uptake of ^99m^Tc-albumin into the olfactory bulb or hypothalamus over time (data not shown). These data show there is no BBB disruption to albumin and the whole brain vascular space is not impacted by CNS treatment with the doses of insulin or S961 used in the time frame investigated.

### Effect of CNS treatment on ^125^I-insulin BBB transport

**Fig. 4:**
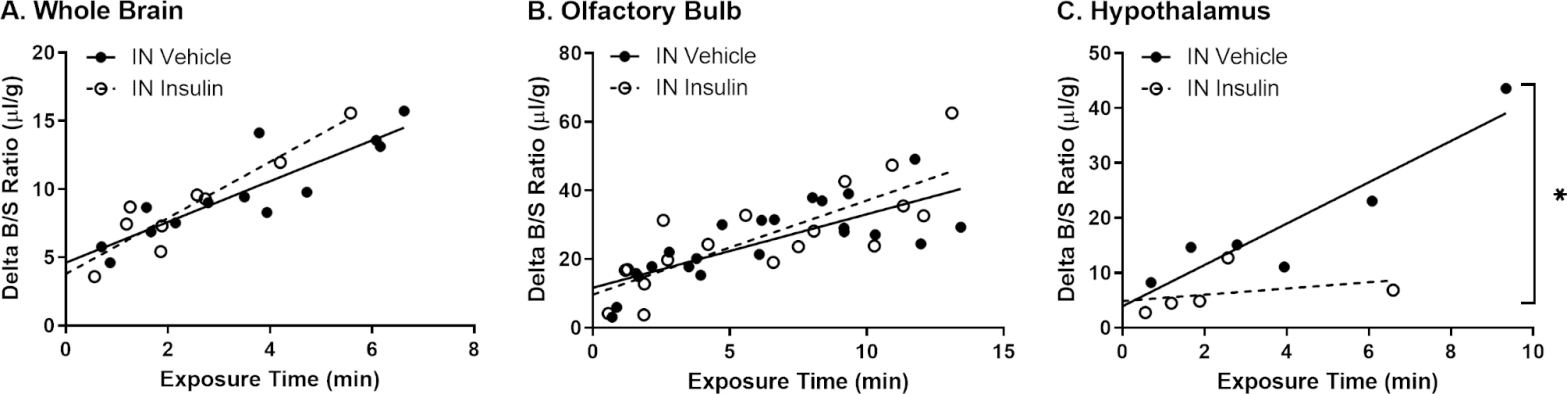
^125^I-insulin blood-to-brain transport following intranasal insulin. ^125^I-insulin BBB pharmacokinetics were assessed 30 min after 1 µl IN injection of vehicle (1% BSA/LR) or insulin (1 µg) in the **(A)** whole brain, **(B)** olfactory bulb, and **(C)** hypothalamus. See Table 1 for pharmacokinetic calculations. Each data point represents a single mouse. K_i_ *p < 0.05 as marked

Next, we investigated the impact of administering insulin or S961 into brain on ^125^I-insulin blood-to-brain transport. There was no effect of IN insulin (30 min) on ^125^I-insulin blood-to-brain transport in the whole brain (IN Vehicle K_*i*_ = 1.49 ± 0.24 vs. IN Insulin K_*i*_ = 2.05 ± 0.31 µl/g-min, p = 0.193) or olfactory bulb (IN Vehicle K_*i*_ = 2.14 ± 0.41 vs. IN Insulin K_*i*_ = 2.74 ± 0.52 µl/g-min, p = 0.366) (Fig. 4A, B; Table 1). However, in a follow up study, IN insulin slowed the blood-to-brain transport rate of ^125^I-insulin into the hypothalamus by about 85% (IN Vehicle K_*i*_ = 3.76 ± 0.76 vs. IN Insulin K_*i*_ = 0.57 ± 0.88 (ns) µl/g-min, p = 0.037) (Fig. 4C; Table 1). These data suggest IN insulin delivery regionally regulates the blood-to-brain transport system for insulin.

**Table 1:** ^125^I-insulin blood-to-brain pharmacokinetics following intranasal insulin

Brain	Group	K_*i*_ (µl/g-min)	p	r	V_*i*_ (µl/g)
Whole Brain	Vehicle	1.49 ± 0.24	0.193	0.89	4.6 ± 0.9
	Insulin	2.05 ± 0.31		0.93	3.8 ± 0.9
Olfactory Bulb	Vehicle	2.14 ± 0.41	0.366	0.76	11.7 ± 2.9
	Insulin	2.74 ± 0.52		0.79	9.7 ± 3.9
Hypothalamus	Vehicle	3.76 ± 0.76	0.037	0.93	3.9 ± 3.8
	Insulin	0.57 ± 0.88 (ns)		0.35	4.9 ± 2.9

Data in this table are generated from the analysis of the graphs in Fig. 4

**Fig. 5:**
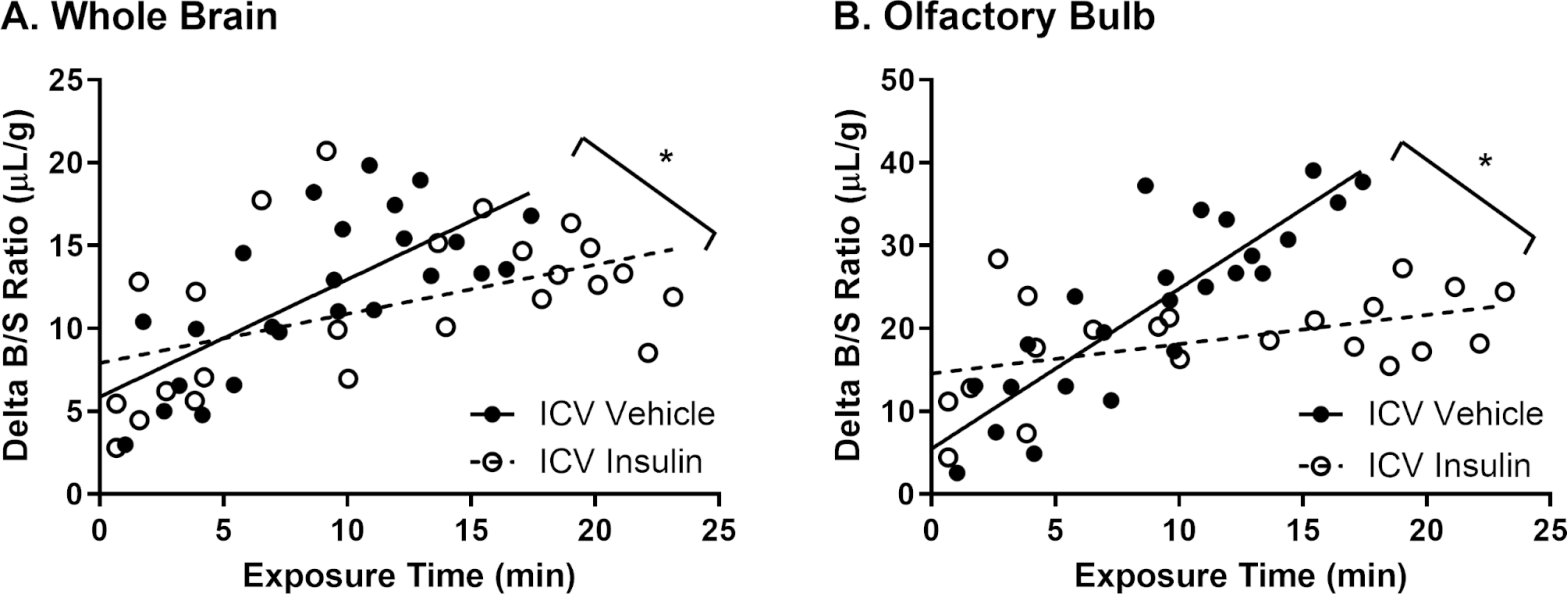
^125^I-insulin blood-to-brain transport following ICV insulin. ^125^I-insulin BBB pharmacokinetics were assessed 10 min after 1 µl ICV injection of vehicle (PB) or insulin (1 µg) in the **(A)** whole brain and **(B)** olfactory bulb. See Table 2 for pharmacokinetic calculations. Each data point represents a single mouse. K_i_ *p < 0.05 as marked

When we investigated the impact of delivering insulin to the CNS via ICV delivery (10 min), we found the rate of transport of ^125^I-insulin across the BBB from blood-to-brain was decreased by about 57% in the whole brain (ICV Vehicle K_*i*_ = 0.71 ± 0.14 vs. ICV Insulin K_*i*_ = 0.30 ± 0.11 µl/g-min, p = 0.037) and by about 82% in the olfactory bulb (ICV Vehicle K_*i*_ = 1.93 ± 0.25 vs. ICV Insulin K_*i*_ = 0.35 ± 0.16 µl/g-min, p < 0.0001) (Fig. 5; Table 2). These data suggest higher insulin levels in the brain can slow the blood-to-brain transport system for insulin.

**Table 2:** ^125^I-insulin blood-to-brain pharmacokinetics following ICV insulin

Brain	Group	K_*i*_ (µl/g-min)	p	r	V_*i*_ (µl/g)
Whole Brain	Vehicle	0.71 ± 0.14	0.037	0.72	5.9 ± 1.5
	Insulin	0.30 ± 0.11		0.50	7.9 ± 1.5
Olfactory Bulb	Vehicle	1.93 ± 0.25	< 0.0001	0.86	5.5 ± 2.5
	Insulin	0.35 ± 0.16		0.45	14.6 ± 2.2

Data in this table are generated from the analysis of the graphs in Fig. 5

**Fig. 6:**
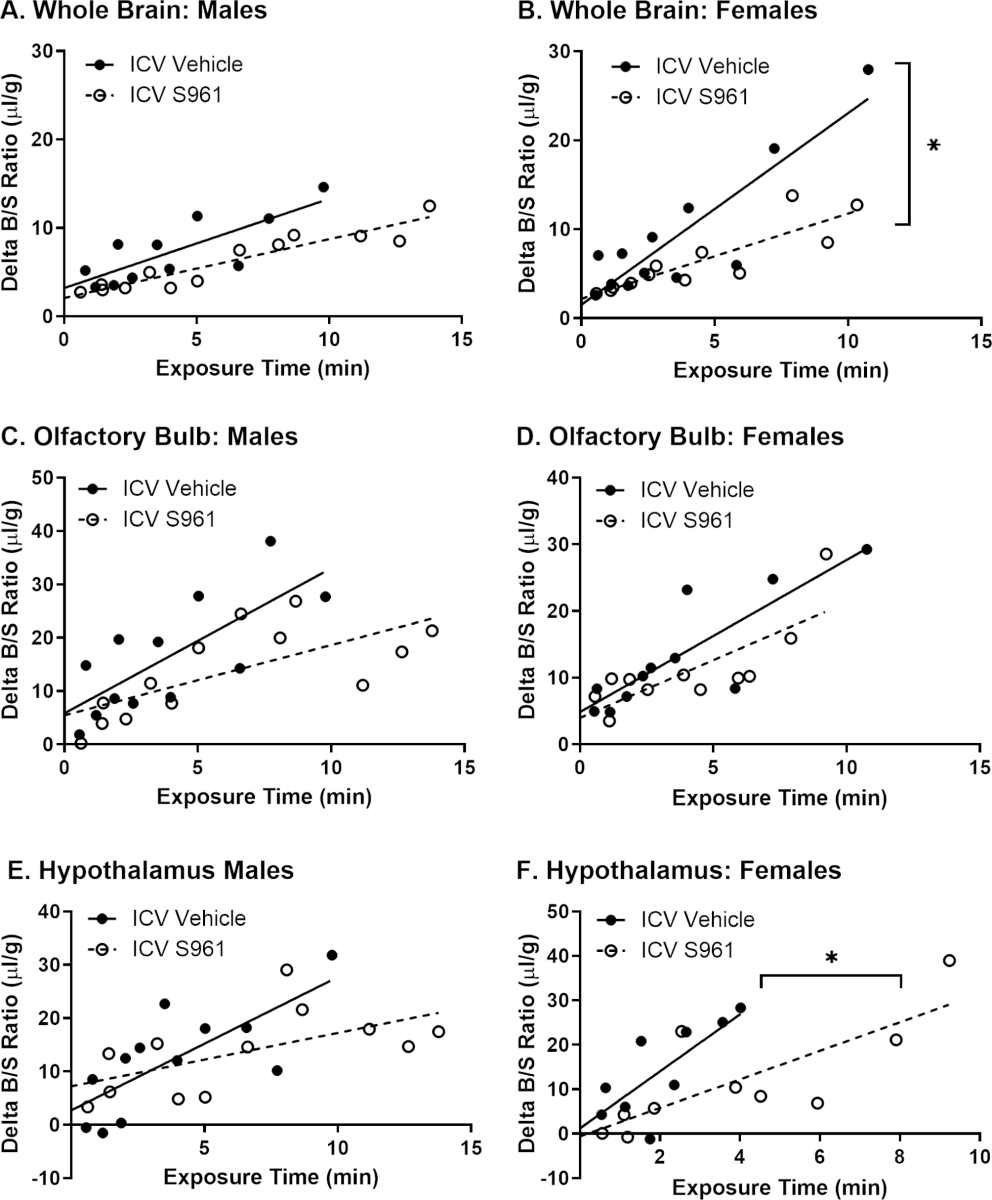
^125^I-insulin blood-to-brain transport following ICV S961. ^125^I-insulin BBB pharmacokinetics were assessed 10 min after 1 µl ICV injection of vehicle (PB) or S961 (1 µg) in the **A/B**) whole brain, **C/D**) olfactory bulb, and E/F) hypothalamus in males **(A, C, E)** and females **(B, D, F)**. See Table 3 for pharmacokinetic calculations. Each data point represents a single mouse. K_i_ *p < 0.05 as marked

We then blocked CNS insulin action as a model of CNS insulin resistance by injecting S961 ICV. We found ICV S961 slows the rate of transport of ^125^I-insulin from blood-to-brain by about 55% in the whole brain only in females (ICV Vehicle K_*i*_ = 2.15 ± 0.36 vs. ICV S961 K_*i*_ = 0.96 ± 0.17 µl/g-min, p = 0.007) (Fig. 6B; Table 3). ICV S961 reduced the amount of vascular binding (V_*i*_) for ^125^I-insulin to about 44% in male whole brain (ICV Vehicle V_*i*_ = 3.2 ± 1.3 vs. ICV S961 V_*i*_ = 2.1 ± 0.6 µl/g, p = 0.002) (Fig. 6A; Table 3). There was no impact of ICV S961 of the rate of ^125^I-insulin blood-to-brain transport in the olfactory bulb or hypothalamus in males or females (Fig. 6C-F; Table 3), although there was a trend towards a decrease in the transport rate to 40% into the hypothalamus in males (p = 0.084). Vascular binding of ^125^I-insulin was decreased in the olfactory bulb in males (ICV Vehicle V_*i*_ = 5.8 ± 3.7 vs. ICV S961 V_*i*_ = 5.5 ± 3.0 µl/g, p = 0.040). These data suggest CNS insulin resistance can regulate the blood-to-brain transport system and interactions for insulin at the BBB in a sex-specific manner and may be regionally regulated.

**Table 3:** ^125^I-insulin blood-to-brain pharmacokinetics following ICV S961

Brain Region	Sex	Group	K_*i*_ (µl/g-min)	K_*i*_ p	r	V_*i*_ (µl/g)	V_*i*_ p	
Whole Brain	Male	Vehicle	1.01 ± 0.26	0.143	0.80	3.2 ± 1.3	0.002	
		S961	0.66 ± 0.07		0.94	2.1 ± 0.6		
	Female	Vehicle	2.15 ± 0.36	0.007	0.88	1.5 ± 1.7	-	
		S961	0.96 ± 0.17		0.87	2.2 ± 0.92		
Olfactory Bulb	Male	Vehicle	2.72 ± 0.78	0.111	0.74	5.8 ± 3.7	0.040	
		S961	1.32 ± 0.41		0.70	5.5 ± 3.0		
	Female	Vehicle	2.28 ± 0.48	0.418	0.85	4.9 ± 2.3	0.131	
		S961	1.73 ± 0.46		0.78	4.0 ± 2.3		
Hypothalamus	Male	Vehicle	2.50 ± 0.71	0.084	0.74	2.7 ± 3.4	0.455	
		S961	1.00 ± 0.43		0.59	7.2 ± 3.4		
	Female	Vehicle	6.42 ± 2.08	0.200	0.76	1.2 ± 4.8	0.036	
		S961	3.21 ± 0.91		0.78	-0.6 ± 4.4		

Data in this table are generated from the analysis of the graphs in Fig. 6

### Effect of CNS treatment on serum insulin levels

**Fig. 7:**
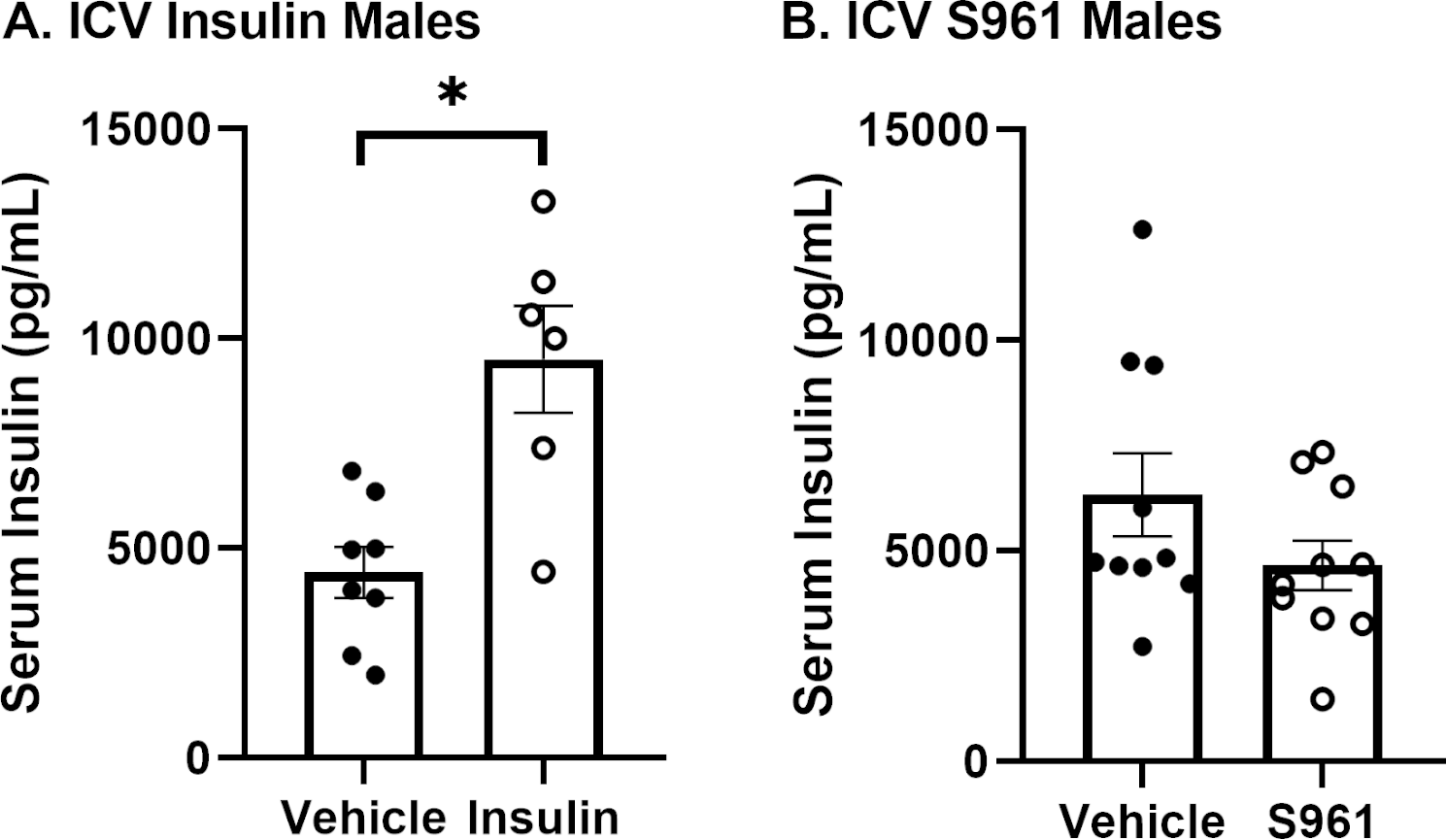
Serum insulin levels following CNS treatment. 30 min following **(A)** ICV insulin, there was a significant increase in the levels of serum insulin but not following **(B)** ICV S961. Each data point represents a single mouse. Student’s t-test *p < 0.01 as marked

As CNS insulin signaling is known to affect peripheral insulin levels, we wanted to identify whether CNS insulin or S961 was altering endogenous mouse serum insulin levels. Insulin transport is saturable [11, 12] so changes in serum insulin levels could impact ^125^I-insulin BBB transport. Thirty minutes following ICV vehicle, human insulin (1 µg), or S961 (1 µg) delivery in male mice, serum was collected and assayed for mouse insulin. However, upon further correspondence with the kit manufacturer, it was noted this kit cross reacts with human insulin. CNS insulin delivery nearly doubled the amount of insulin present in the serum (Fig. 7A). We cannot conclude from this data whether CNS insulin affects endogenous peripheral insulin levels or enters the circulation following ICV injection, as shown in Fig. 1A. CNS S961 had no effect on serum insulin levels 30 min following treatment (Fig. 7B). These data suggest CNS insulin receptor inhibition does not impact peripheral insulin levels in the time frame investigated.

### Effect of CNS insulin resistance on IN ^125^I-insulin distribution

**Fig. 8:**
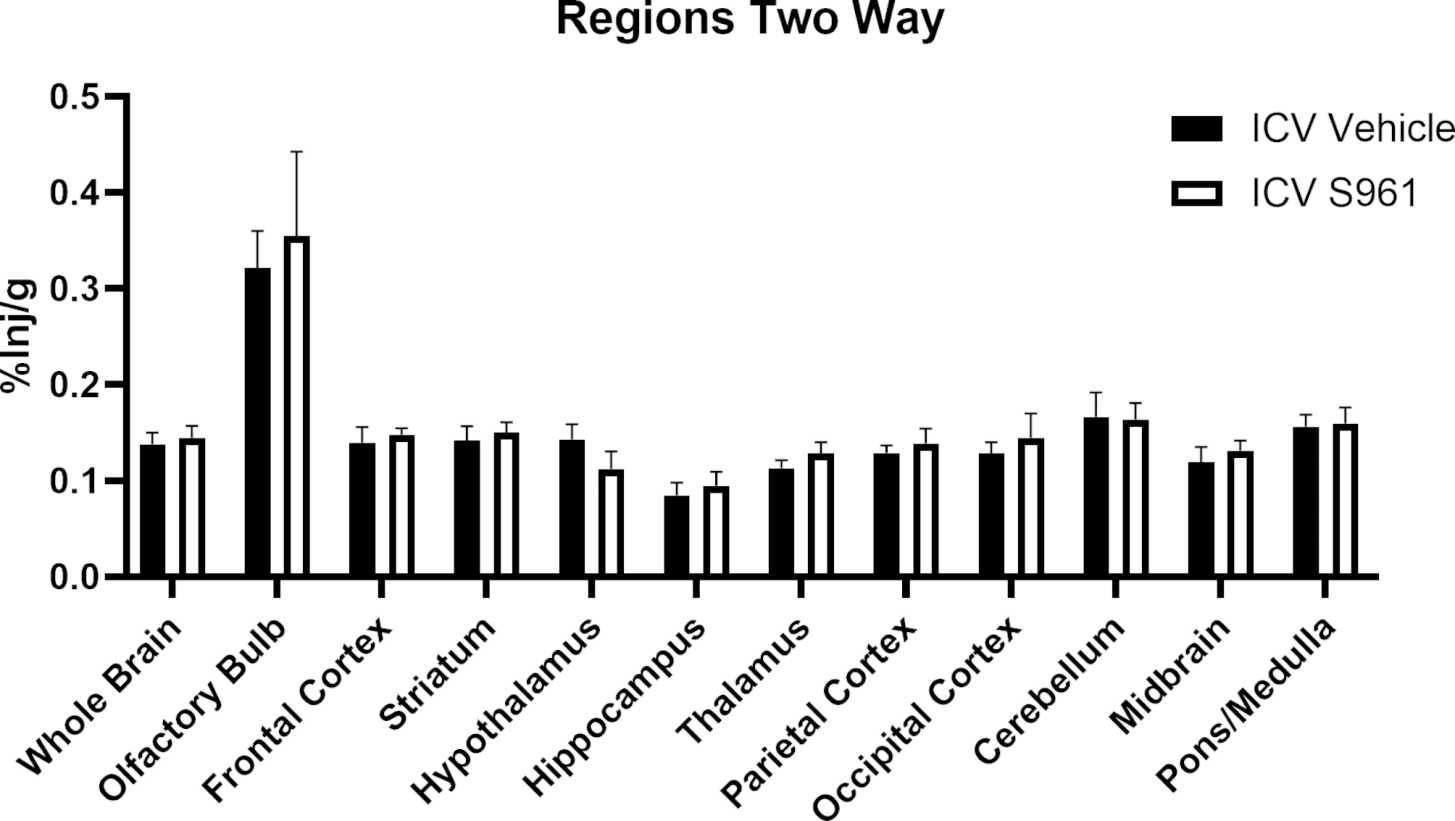
IN ^125^I-insulin brain distribution following ICV S961. There were no statistical differences (two-way ANOVA) in regional ^125^I-insulin levels (%Inj/g) following ICV S961 (n = 4/group)

IN insulin is currently being investigated in the clinic as a therapy to delay cognitive decline [25] in patients that likely have CNS insulin resistance [33]. We wanted to verify CNS insulin resistance, via CNS insulin receptor blockade using S961, did not affect distribution of ^125^I-insulin following IN delivery. Immediately following ICV administration of vehicle or S961 (1 µg), we measured regional distribution of ^125^I-insulin following IN administration. We found ICV S961 did not impact ^125^I-insulin brain distribution after 30 min (Fig. 8), similar to previous findings in a mouse model of sporadic AD [30]. This data suggests CNS insulin resistance does not affect transport and distribution of ^125^I-insulin following IN delivery.

### Effect of CNS treatment on regional vascular space

**Fig. 9:**
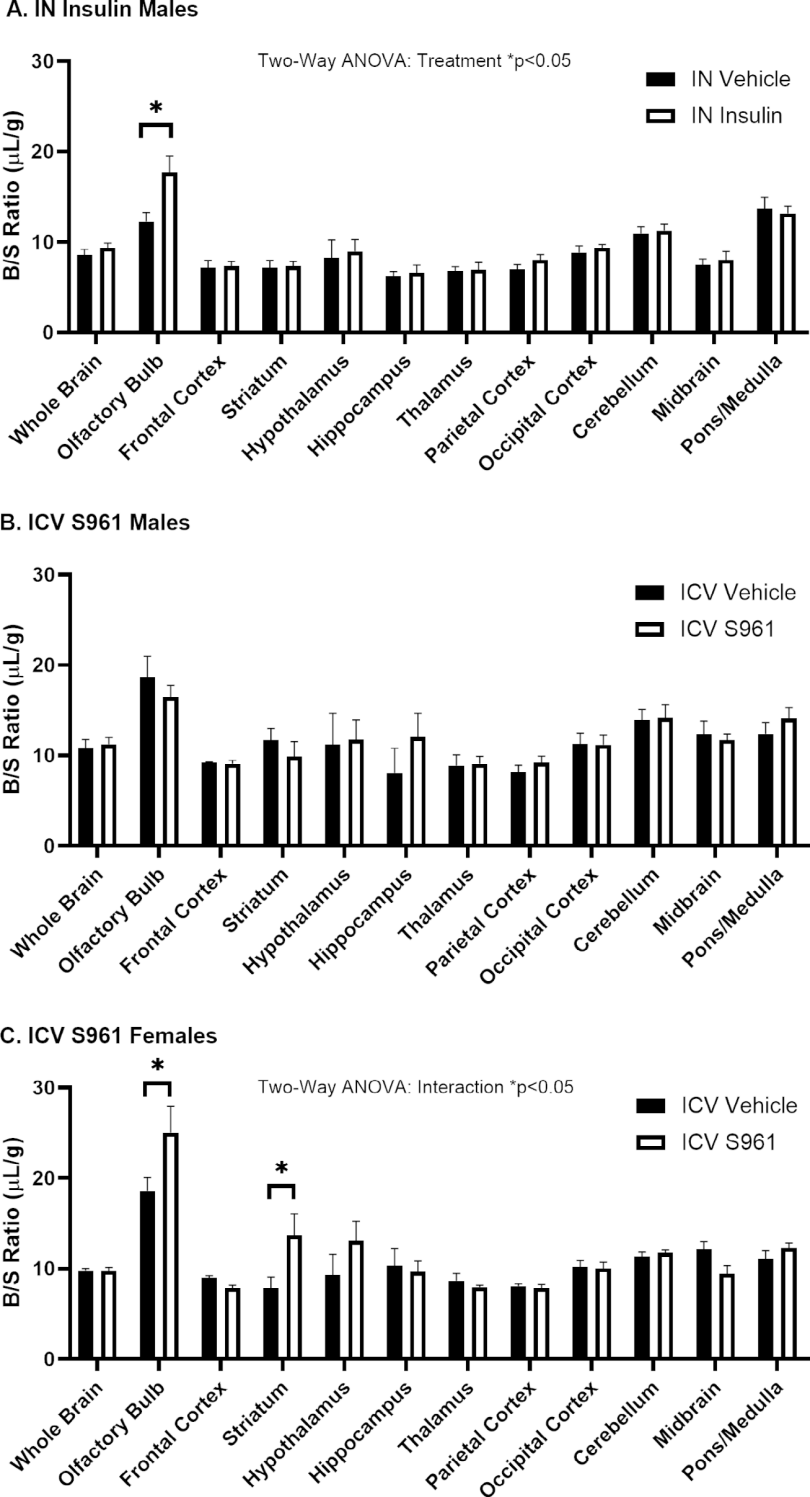
^99m^Tc-albumin blood-to-brain regional levels following CNS treatment. **(A)** IN insulin in males significantly affects ^99m^Tc-albumin regional levels (two-way ANOVA: treatment p < 0.05; post hoc *p < 0.05 as marked, n = 5–6/group). **(B)** ICV S961 in males does not affect ^99m^Tc-albumin regional levels (two-way ANOVA: treatment p = 0.673; n = 4–6/group). **(C)** ICV S961 in females significantly affects ^99m^Tc-albumin levels(two-way ANOVA: interaction p < 0.05; treatment p = 0.056; Tukey’s post hoc *p < 0.05 as marked, n = 5–6/group)

Due to a potential regional regulation of IN insulin or ICV S961 on ^125^I-insulin BBB transport, we performed follow-up studies on various regions throughout the brain. We correct for vascular space using ^99m^Tc-albumin when measuring ^125^I-insulin brain uptake. ^99m^Tc-albumin does not cross the BBB in the time frame investigated (Fig. 3). Therefore, any changes in ^99m^Tc-albumin B/S levels would be attributed to changes in the vascular space. When we calculated the B/S levels of ^99m^Tc-albumin, we found treatment differences in select regions both following IN insulin and ICV S961 administration. Thirty min following IN insulin (1 µg) in male mice, we saw a 43% increase in the amount of ^99m^Tc-albumin in the olfactory bulb (IN Vehicle B/S ratio = 12.3 ± 1.0 vs. IN Insulin B/S ratio = 17.7 ± 1.8 µL/g, p = 0.023) (Fig. 9A). Ten min following ICV S961 (1 µg), we observed no difference in male mice (Fig. 9B). However, we saw a 34% increase in the amount of ^99m^Tc-albumin in the female olfactory bulb (ICV Vehicle B/S ratio = 18.6 ± 1.5 vs. ICV S961 B/S ratio = 25.1 ± 2.9 µL/g, p = 0.008) and a 76% increase in the striatum (ICV Vehicle B/S ratio = 7.8 ± 1.2 µL/g vs. ICV S961 B/S ratio = 13.7 ± 2.4 µL/g, p = 0.024) following ICV S961 administration (Fig. 9C). These data suggest CNS insulin signaling can impact regional vascular space, particularly in the olfactory bulb.

### Effect of CNS treatment on regional ^125^I-insulin brain uptake

**Fig. 10:**
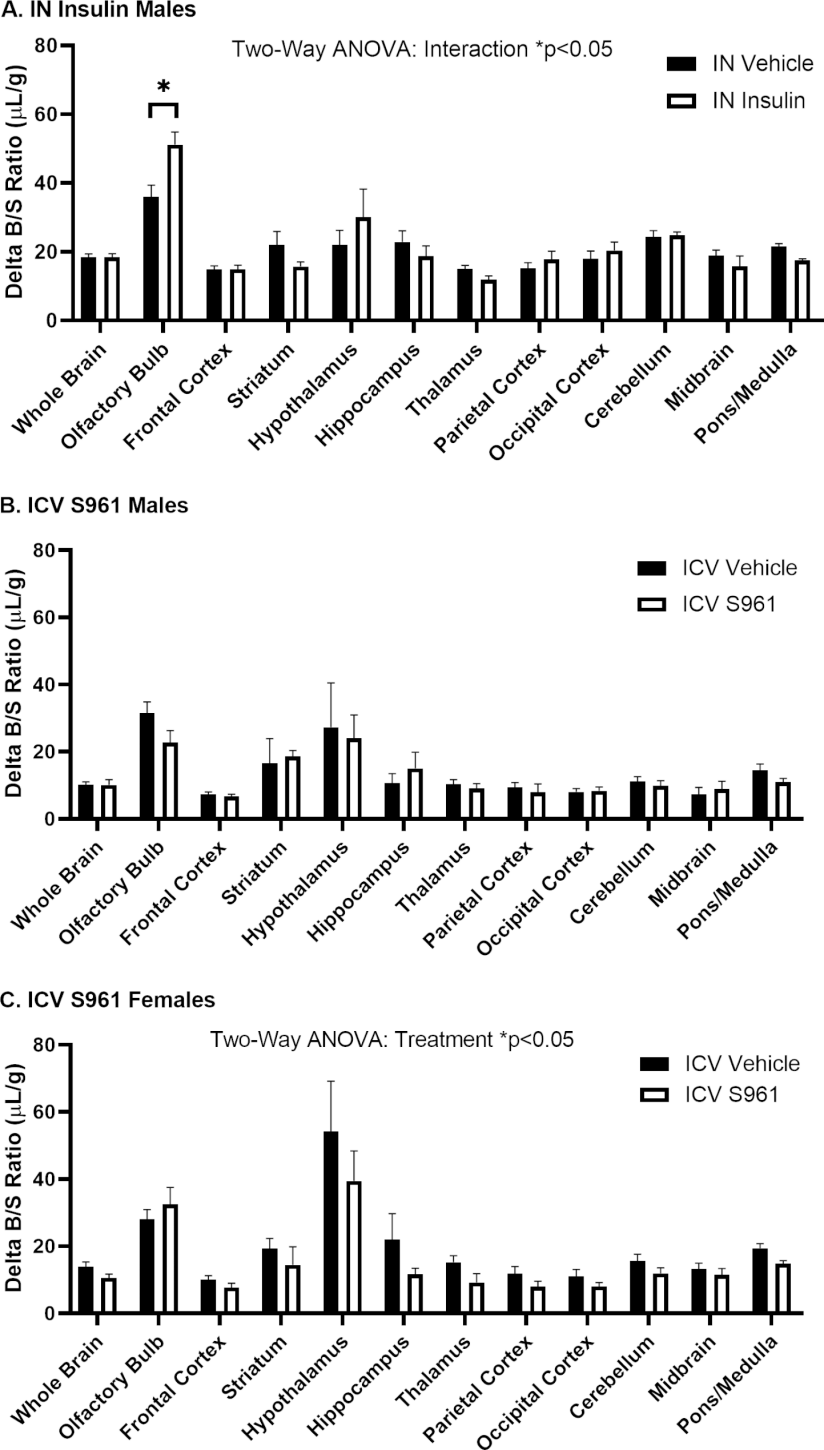
^125^I-insulin blood-to-brain regional uptake following CNS treatment. ^125^I-insulin regional uptake was **(A)** significantly affected by IN insulin in males (two-way ANOVA: interaction p < 0.05; Tukey’s post hoc *p < 0.05 as marked, n = 5–6/group) and **(B)** ICV S961 in males (two-way ANOVA: treatment p = 0.476; n = 4–6/group) but was significantly affected by **(C)** ICV S961 in females (two-way ANOVA: treatment p < 0.05, n = 5–6/group)

Lastly, we identified whether IN insulin or ICV S961 regulates ^125^I-insulin brain uptake regionally. Thirty min following IN insulin in male mice, we found overall differences in ^125^I-insulin regional uptake (two-way ANOVA, interaction p = 0.0157), after correction for vascular space (Delta B/S ratios). Specifically, there was an increase in the amount of ^125^I-insulin present in the olfactory bulb (IN vehicle delta B/S ratio 36.1 ± 3.3 µL/g vs. IN insulin delta B/S ratio = 51.2 ± 3.7 µL/g, p = 0.0025) (Fig. 10A). Ten min following ICV S961 in male mice, there were no regional differences in ^125^I-insulin brain uptake (Fig. 10B). However, following ICV S961 in female mice (Fig. 10C), we found decreases in the amount of insulin uptake (two-way ANOVA, treatment p = 0.0101) with no post hoc differences, consistent with our data showing decreased insulin BBB transport in female mice following ICV S961. These data suggest CNS insulin signaling can broadly impact ^125^I-insulin brain uptake.

## Discussion

We have previously shown genetic knock-down of the endothelial insulin receptor or pharmacological inhibition of the endothelial receptor using S961 did not affect the transport of insulin across the BBB [20]. We wanted to extend these findings to the CNS to determine whether the insulin receptor present in CNS cell types could regulate insulin BBB transport. In this study, we investigated whether the brain could control its own rate of insulin entry from the periphery, across the BBB. Specifically, we investigated whether brain insulin concentrations or insulin receptor signaling inhibition could regulate insulin BBB transport. In time frames where we know our CNS manipulations (insulin or the insulin receptor antagonist, S961) can distribute throughout the brain, we investigated radioactive insulin BBB interactions. We found IN insulin did not have an effect on insulin whole brain or olfactory bulb BBB transport but did decrease transport into the hypothalamus. ICV insulin significantly decreased whole brain and olfactory bulb insulin BBB transport. Lastly, we found CNS insulin resistance, as defined by impaired insulin signaling through administration of ICV S961, decreased insulin BBB interactions in both male (binding) and female (transport) mice and that these effects may be regionally regulated. These data support a central feedback mechanism to the BBB in regulating insulin entry into the brain.

We found following ICV delivery of ^125^I-insulin, a small amount enters the blood (< 3%Inj/ml). We found both insulin and S961 (a 43-amino acid single chain peptide) distribute throughout the brain following ICV delivery. We found differing effects on insulin blood-to-brain transport following IN insulin compared to ICV insulin. We believe these differing effects are due to discrepancies in the amounts of insulin reaching critical brain regions. We know from our previous study that approximately 0.13%Inj/g of ^125^I-insulin is present in the whole brain 30 min after IN delivery in CD-1 male mice [30]. Therefore, when we give a 1 µg IN administration, we would expect approximately 1.3 ng per gram of brain tissue to be present. As the brain weighs approximately 0.5 g, there is approximately 0.65 ng insulin present in the entire brain following IN administration. In comparison, due to the direct delivery to the brain, following ICV delivery, we observed approximately 60.5%Inj/g insulin and 92.6%Inj/g S961 in the whole brain at 10 min. Therefore, when we give a 1 µg ICV administration, we would expect approximately 605 ng insulin and 926 ng S961 per gram of brain tissue to be present. To summarize, ICV delivery results in a much greater amount of insulin in the brain compared to IN delivery. These calculations are important for the interpretation of the insulin BBB transport findings as described next.

We found IN insulin was unable to affect ^125^I-insulin BBB transport or binding in whole brain and the olfactory bulb. However, we found this regulation was regionally mediated. Transport into the hypothalamus was decreased nearly 7-fold in response to 1 µg IN insulin. Our previous study showed that by 30 min, the hypothalamus had the third highest (only olfactory bulb and midbrain had higher levels) amount of insulin present (0.23%Inj/g) following IN insulin delivery in male CD-1 mice [30]. Since the transport rate into the olfactory bulb was not affected, our data suggest the hypothalamus is much more sensitive to small changes in insulin concentration and may be a region of the brain where very little insulin needs to be present to induce a large downstream effect.

In comparison, high levels of insulin delivered into the brain via ICV administration significantly decreased ^125^I-insulin BBB transport. In both the whole brain and olfactory bulb, transport rates were decreased to nearly the same rate, regardless of the initial rate (i.e., the vehicle group). These data not only support a negative feedback loop for CNS levels on BBB function but also suggest there could be a floor effect in this loop, since both the whole brain and olfactory bulb were reduced to the same K_*i*_ (~ 0.3 µl/g-min).

Most importantly, we wanted to investigate whether deficiencies in CNS insulin signaling, as occurs in CNS insulin resistance, regulated insulin BBB transport. In these studies, we observed sex differences in the response to ICV S961. Females were more sensitive to CNS insulin receptor inhibition and significantly decreased the whole brain ^125^I-insulin BBB transport rate. While males did not exhibit a decrease in the transport rate, there were significant decreases in the level of reversible vascular binding (V_*i*_) in the whole brain. We did not perform ^125^I-S961 brain distribution studies in females. It is possible S961 is able to spread more throughout the female brain compared to males, affecting a greater number of CNS insulin receptors. However, it is more likely there are differences in the insulin receptor signaling pathways due to sex, as has been shown numerous times in response to insulin administration [34–37]. Additionally, as females are more at risk for developing AD, the sensitivity to CNS insulin resistance, a feature of AD, in relation to insulin BBB transport could be a reason why females are at a greater risk. While a direct link between insulin BBB transport and CNS insulin resistance has not been established, this data points to a connection between insulin BBB transport and the development of CNS insulin resistance. In the olfactory bulb, there were significant decreases in ^125^I-insulin BBB binding in males only and the hypothalamus was too variable to observe any significant differences.

Since there was no effect on whole brain insulin BBB transport following IN insulin delivery but there was a significant decrease in insulin BBB transport following ICV insulin delivery, we attribute these differences due to the amount of insulin acting within the brain. There are two important observations from these findings. First, therapeutic delivery of IN insulin is likely not impacting endogenous whole brain insulin BBB transport. Second, if insulin concentrations get high enough within the brain, there is a negative feedback loop on BBB transport. Follow-up studies will need to be performed to test varying doses of ICV insulin and identify the lowest concentration of insulin in the CSF that is necessary to negatively impact insulin BBB transport.

One way the insulin BBB transport rate for ^125^I-insulin can be decreased is by an increase in endogenous serum insulin level since this transport system is saturable [11], and the endogenous insulin would compete with the ^125^I-insulin for transport. We observe about a 50% decrease in ^125^I-insulin BBB transport rates in whole brain following ICV insulin, while also observing a two-fold increase in serum insulin levels. While we are not able to discriminate between mouse and human insulin using this kit, our data from the ICV ^125^I-insulin distribution study shows us approximately 2% of what is injected ICV enters per ml of serum.

Previous studies by our group have shown an IV bolus of 121 ng unlabeled insulin per mouse is necessary to reduce the ^125^I-insulin BBB Ki by 50% [11]. The average blood volume of a mouse is about 77–80 ml/kg [38, 39]. Given that our male CD-1 mice are approximately 30 g at the time of study, these mice have around 2.4 ml of blood. A 121 ng injection of insulin would result in a 50.4 ng/ml blood concentration. In our study, following 1 µg human insulin ICV delivery in males, serum insulin levels rise 5 ng/ml, likely due to the appearance of human insulin in the blood. Therefore, while the increase in serum insulin is significant compared to vehicle treated animals, it is about 10-fold too little to account for the decreased ^125^I-insulin BBB transport rates. It should also be noted previous studies show acute ICV insulin injections can increase plasma insulin levels within 5-120 min after injection [40, 41]. However, to confirm whether the increased levels of serum insulin we detected are due to exogenous human insulin or endogenous pancreatic release of mouse insulin, further studies would need to be performed with more sensitive assays. ICV S961 does not impact serum insulin levels, suggesting inhibition of brain insulin signaling does not conversely impact serum insulin levels within the 30 min time frame and the decrease observed in insulin blood-to-brain transport was not due to serum insulin changes. IN insulin also does not significantly impact serum insulin levels [42, 43].

Next, we wanted to identify whether impairments in CNS insulin receptor signaling would affect the ability of the brain to take up ^125^I-insulin following IN delivery. This is important as IN insulin is being pursued in the clinic for the prevention of cognitive decline [25]. CNS insulin resistance negatively correlates with cognition [33]. Therefore, we wanted to verify the presence of CNS insulin resistance, via ICV delivery of S961, did not affect the distribution of IN insulin. The results were positive, with no significant changes in the brain distribution of ^125^I-insulin following S961 treatment. These data suggest CNS insulin resistance, as occurs in AD [33], is not likely to impact the therapeutic delivery of IN insulin, similar to what we have previously shown in the SAMP8 mouse model of AD [30].

Due to the potential regional regulation of insulin BBB transport following CNS treatment with insulin or S961, we wanted to identify whether there were, in fact, differences in the regional uptake of ^125^I-insulin. We first measured the vascular space in each region using the ^99m^Tc-albumin B/S ratios. ^99m^Tc-albumin does not cross the BBB in this time frame under normal conditions. However, we found similar increases in the olfactory bulb following IN insulin in male mice and following ICV S961 in female mice. This is the first report we are aware of showing increased vascular space in the olfactory bulb following CNS activation or inhibition of insulin signaling. This was surprising to us as we have not observed differences in vascular space in previous studies investigating insulin BBB transport in various models [20, 44, 45], despite evidence showing the olfactory bulb has the fastest rate of insulin BBB transport [17]. Possible explanations for the increased level of ^99m^Tc-albumin could be (1) increased ^99m^Tc-albumin leakage across the BBB, (2) vasodilation in the brain regions, or (3) a decrease in the brain capillary hematocrit [46]. The middle explanation is the most plausible, as previous studies have shown a role for insulin signaling in regulating vascular reactivity, due to the involvement in nitric oxide signaling [47]. CNS insulin can increase sympathetic nerve activity which could affect vasodilation in the periphery [48, 49]. There is also a cerebrovascular response to insulin [50]. In isolated rat cerebral arteries, 10 min exposure to a range of insulin concentrations resulted in a dose response change in vasodilation. These findings were translated to increased cortical blood flow in vivo following exposure to insulin. Pericytes can regulate brain capillary diameter [51]. Therefore, it is possible insulin signaling is critical in this region and manipulations in this pathway has consequences, particularly on vascular space.

Finally, we identified regional differences in ^125^I-insulin uptake in response to CNS insulin and S961. Instead of looking at transport rate as in our earlier studies, we measured ^125^I-insulin delta B/S ratios (i.e., brain/serum ratios corrected for brain vascular space) at a single time point, 5 min, a time when ^125^I-insulin BBB transport is in its linear phase. Following IN insulin delivery, there is a significant treatment effect in the delta B/S ratio of ^125^I-insulin. The olfactory bulb had significantly increased delta B/S values following IN insulin. This suggests there is either increased ^125^I-insulin BBB binding or increased transport. In our pharmacokinetic studies (Fig. 4), we showed there was no significant difference in the whole brain transport rate. However, at a single time point in the middle of the transport curve, IN insulin delta B/S ratios are greater than the IN vehicle values. This suggests there is more likely an increase in the amount of ^125^I-insulin binding in this region which could translate to an increase in insulin BBB signaling. Similar to our pharmacokinetic data showing a decreased ^125^I-insulin BBB transport rate in response to ICV S961 in females, our regional data showed a significant treatment effect, with mostly decreased ^125^I-insulin levels across all regions (no post hoc differences). These data suggest global brain insulin resistance does, in fact, affect global insulin BBB transport. We are working on follow-up studies to identify whether CNS insulin resistance in select brain regions impacts regional or global insulin BBB transport.

There are some limitations of our studies we would like to address. First, we investigated the impact of ICV insulin on BBB transport. We do not know what impact perivascular levels or even parenchymal levels of insulin would have on BBB transport. Another limitation is that all of our studies were acute (< 30 min). We are not able to address from our study what impact chronically high CNS levels of insulin would have on BBB transport. We predict chronically elevated levels would show more robust changes. Lastly, we only used one concentration of insulin or S961 delivered to the brain. As disruption of brain insulin signaling in AD is likely a spectrum, further dose-response studies are warranted.

## Conclusion

Overall, our findings show high concentrations of brain insulin can slow the BBB transport system for insulin while loss of signaling altogether impairs the ability for insulin to cross the BBB. IN insulin does not affect whole brain insulin uptake but may impair hypothalamic insulin transport and enhance olfactory bulb insulin uptake. ICV insulin has a much greater impairment on whole brain and olfactory bulb insulin transport, likely because ICV administration of insulin increases brain levels much more than does IN administration. CNS insulin resistance appears to negatively influence whole brain insulin transport and overall regional uptake more greatly in females. These data for the first time show the brain can influence the rate of insulin transport across the BBB in the blood-to-brain direction. As brain insulin resistance is a common feature of those with dementia, including AD, it further supports the role of the BBB in brain insulin resistance.

## Data Availability

The datasets supporting the conclusions of this article are included within the article.

## Abbreviations

BBB: blood-brain barrier
AD: Alzheimer’s disease
IN: intranasal
ICV: intracerebroventricular
BSA: bovine serum albumin
LR: lactated Ringer’s
CPM: counts per minute
IV: intravenous
PB: phosphate buffer
MTRA: multiple-time regression analysis
B/S: brain/serum
OB: olfactory bulb
St: striatum
FC: frontal cortex
Hy: hypothalamus
HC: hippocampus
Th: thalamus
PC: parietal cortex
OC: occipital cortex
CB: cerebellum
MB: midbrain
PM: pons-medulla
WB: whole brain
CNS: central nervous system,

## References

[1] Banks WA, Owen JB, Erickson MA (2012). Insulin in the brain: there and back again. Pharmacol Ther.

[2] Rhea EM, Banks WA (2019). Role of the blood-brain barrier in central nervous system insulin resistance. Front NeuroSci.

[3] Stanley M, Macauley SL, Caesar EE, Koscal LJ, Moritz W, Robinson GO (2016). The Effects of Peripheral and Central High insulin on brain insulin signaling and amyloid-beta in Young and Old APP/PS1 mice. J Neurosci.

[4] Craft S, Peskind E, Schwartz MW, Schellenberg GD, Raskind M, Porte D (1998). Jr. Cerebrospinal fluid and plasma insulin levels in Alzheimer’s disease: relationship to severity of dementia and apolipoprotein E genotype. Neurology.

[5] Bromander S, Anckarsater R, Ahren B, Kristiansson M, Blennow K, Holmang A (2010). Cerebrospinal fluid insulin during non-neurological surgery. J Neural Transm (Vienna).

[6] Rhea EM, Banks WA (2021). A historical perspective on the interactions of insulin at the blood-brain barrier. J Neuroendocrinol.

[7] Baskin DG, Porte D, Guest K, Dorsa DM (1983). Regional concentrations of insulin in the rat brain. Endocrinology.

[8] Mazucanti CH, Liu QR, Lang D, Huang N, O’Connell JF, Camandola S et al. Release of insulin produced by the choroid plexis is regulated by serotonergic signaling.JCI Insight. 2019;4(23). 10.1172/jci.insight.131682 PMC6962018 31647782

[9] Sylow L, Tokarz VL, Richter EA, Klip A (2021). The many actions of insulin in skeletal muscle, the paramount tissue determining glycemia. Cell Metab.

[10] Banks WAN, Rhea C. E. M. Evidence for an alternative insulin transporter at the bloodbrain barrier.Aging Patholobiology and Therapeutics. 2022;4(4). 10.31491/apt.2022.12.100 PMC9837797 36644126

[11] Banks WA, Jaspan JB, Huang W, Kastin AJ (1997). Transport of insulin across the blood-brain barrier: saturability at euglycemic doses of insulin. Peptides.

[12] Baura GD, Foster DM, Porte D, Kahn SE, Bergman RN, Cobelli C (1993). Saturable transport of insulin from plasma into the central nervous system of dogs in vivo. A mechanism for regulated insulin delivery to the brain. J Clin Investig.

[13] Cashion MF, Banks WA, Kastin AJ (1996). Sequestration of centrally administered insulin by the brain: effects of starvation, aluminum, and TNF-alpha. Horm Behav.

[14] Banks WA, Kastin AJ, Huang W, Jaspan JB, Maness LM (1996). Leptin enters the brain by a saturable system independent of insulin. Peptides.

[15] Banks WA, Jaspan JB, Kastin AJ (1997). Selective, physiological transport of insulin across the blood-brain barrier: novel demonstration by species-specific radioimmunoassays. Peptides.

[16] Heni M, Schopfer P, Peter A, Sartorius T, Fritsche A, Synofzik M (2014). Evidence for altered transport of insulin across the blood-brain barrier in insulin-resistant humans. Acta Diabetol.

[17] Banks WA, Kastin AJ, Pan W (1999). Uptake and degradation of blood-borne insulin by the olfactory bulb. Peptides.

[18] Gray SM, Aylor KW, Barrett EJ (2017). Unravelling the regulation of insulin transport across the brain endothelial cell. Diabetologia.

[19] Urayama A, Banks WA (2008). Starvation and triglycerides reverse the obesity-induced impairment of insulin transport at the blood-brain barrier. Endocrinology.

[20] Rhea EM, Rask-Madsen C, Banks WA (2018). Insulin transport across the blood-brain barrier can occur independently of the insulin receptor. J Physiol.

[21] Hersom M, Helms HC, Schmalz C, Pedersen TA, Buckley ST, Brodin B (2018). The insulin receptor is expressed and functional in cultured blood-brain barrier endothelial cells but does not mediate insulin entry from blood to brain. Am J Physiol Endocrinol Metab.

[22] Taubel JC, Nelson NR, Bansal A, Curran GL, Wang L, Wang Z (2022). Design, synthesis, and preliminary evaluation of [(68)Ga]Ga-NOTA-Insulin as a PET probe in an Alzheimer’s Disease Mouse Model. Bioconjug Chem.

[23] Leclerc M, Bourassa P, Tremblay C, Caron V, Sugere C, Emond V et al. Cerebrovascular insulin receptors are defective in Alzheimer’s disease.Brain. 2022. 10.1093/brain/awac309 PMC9897197 36280236

[24] Brown C, Pemberton S, Babin A, Abdulhameed N, Noonan C, Brown MB et al. Insulin blood-brain barrier transport and interactions are greater following exercise in mice. J Appl Physiol (1985). 2022. 10.1152/japplphysiol.00866.2021 PMC8917914 35175106

[25] Craft S, Raman R, Chow TW, Rafii MS, Sun CK, Rissman RA et al. Safety, Efficacy, and Feasibility of Intranasal Insulin for the Treatment of Mild Cognitive Impairment and Alzheimer Disease Dementia: A Randomized Clinical Trial.JAMA neurology. 2020. 10.1001/jamaneurol.2020.1840 PMC7309571 32568367

[26] Rhea EM, Salameh TS, Banks WA (2019). Routes for the delivery of insulin to the central nervous system: a comparative review. Exp Neurol.

[27] Schaffer L, Brand CL, Hansen BF, Ribel U, Shaw AC, Slaaby R (2008). A novel high-affinity peptide antagonist to the insulin receptor. Biochem Biophys Res Commun.

[28] Banks WA, Kastin AJ, Komaki G, Arimura A (1993). Passage of pituitary adenylate cyclase activating polypeptide1-27 and pituitary adenylate cyclase activating polypeptide1-38 across the blood-brain barrier. J Pharmacol Exp Ther.

[29] Banks W, Fasold M, Kastin A. Measurement of Efflux Rates from Brain to blood. In: Irvine GB, Williams C, editors. Neuropeptide Protocols. Methods in Molecular Biology™. Volume 73. Humana Press; 1997. pp. 353–60. 10.1385/0-89603-399-6:353 9031222

[30] Rhea EM, Humann SR, Nirkhe S, Farr SA, Morley JE, Banks WA (2017). Intranasal insulin transport is preserved in aged SAMP8 mice and is altered by albumin and insulin receptor inhibition. J Alzheimers Dis.

[31] Glowinski J, Iversen LL (1966). Regional studies of catecholamines in the rat brain. I. The disposition of [3H]norepinephrine, [3H]dopamine and [3H]dopa in various regions of the brain. J Neurochem.

[32] Zar JH. Biostatistical analysis. 2nd ed. Englewood Cliffs N.J: Prentice-hall; 1984. xiv, 718 p. p.

[33] Talbot K, Wang HY, Kazi H, Han LY, Bakshi KP, Stucky A (2012). Demonstrated brain insulin resistance in Alzheimer’s disease patients is associated with IGF-1 resistance, IRS-1 dysregulation, and cognitive decline. J Clin Investig.

[34] Hallschmid M, Benedict C, Schultes B, Born J, Kern W (2008). Obese men respond to cognitive but not to catabolic brain insulin signaling. Int J Obes (Lond).

[35] Hallschmid M, Benedict C, Schultes B, Fehm HL, Born J, Kern W (2004). Intranasal insulin reduces body fat in men but not in women. Diabetes.

[36] Benedict C, Kern W, Schultes B, Born J, Hallschmid M (2008). Differential sensitivity of men and women to anorexigenic and memory-improving effects of intranasal insulin. J Clin Endocrinol Metab.

[37] Wagner L, Veit R, Fritsche L, Haring HU, Fritsche A, Birkenfeld AL (2022). Sex differences in central insulin action: Effect of intranasal insulin on neural food cue reactivity in adults with normal weight and overweight. Int J Obes (Lond).

[38] Mitruka BMRH (1981). Clinical, biochemical and hematological reference values in normal experimental animals and normal humans.

[39] Harkness JEWJ (1989). The biology and medicine of rabbits and rodents.

[40] Schultz-Klarr S, Wright-Richey J, Dunbar JC (1994). Plasma glucose, insulin and cardiovascular responses after intravenous intracerebroventricular injections of insulin, 2-deoxyglucose and glucose in rats. Diabetes Res Clin Pract.

[41] Chen M, Woods SC, Porte D (1975). Effect of cerebral intraventricular insulin on pancreatic insulin secretion in the dog. Diabetes.

[42] Benedict C, Hallschmid M, Schmitz K, Schultes B, Ratter F, Fehm HL (2007). Intranasal insulin improves memory in humans: superiority of insulin aspart. Neuropsychopharmacology.

[43] Lv H, Tang L, Guo C, Jiang Y, Gao C, Wang Y (2020). Intranasal insulin administration may be highly effective in improving cognitive function in mice with cognitive dysfunction by reversing brain insulin resistance. Cogn Neurodyn.

[44] Rhea EM, Hansen K, Pemberton S, Torres ERS, Holden S, Raber J (2021). Effects of apolipoprotein E isoform, sex, and diet on insulin BBB pharmacokinetics in mice. Sci Rep.

[45] Rhea EM, Torres ERS, Raber J, Banks WA (2020). Insulin BBB pharmacokinetics in young apoE male and female transgenic mice. PLoS ONE.

[46] Hudetz AG (1997). Blood flow in the cerebral capillary network: a review emphasizing observations with intravital microscopy. Microcirculation.

[47] Hill MA, Yang Y, Zhang L, Sun Z, Jia G, Parrish AR (2021). Insulin resistance, cardiovascular stiffening and cardiovascular disease. Metab Clin Exp.

[48] Ward KR, Bardgett JF, Wolfgang L, Stocker SD (2011). Sympathetic response to insulin is mediated by melanocortin 3/4 receptors in the hypothalamic paraventricular nucleus. Hypertension.

[49] Cassaglia PA, Shi Z, Brooks VL (2016). Insulin increases sympathetic nerve activity in part by suppression of tonic inhibitory neuropeptide Y inputs into the paraventricular nucleus in female rats. Am J Physiol Regul Integr Comp Physiol.

[50] Katakam PV, Domoki F, Lenti L, Gaspar T, Institoris A, Snipes JA (2009). Cerebrovascular responses to insulin in rats. J Cereb blood flow metabolism: official J Int Soc Cereb Blood Flow Metabolism.

[51] Hamilton NB, Attwell D, Hall CN. Pericyte-mediated regulation of capillary diameter: a component of neurovascular coupling in health and disease.Front Neuroenergetics. 2010;2. 10.3389/fnene.2010.00005 PMC2912025 20725515

